# The Non-receptor Tyrosine Kinase Pyk2 in Brain Function and Neurological and Psychiatric Diseases

**DOI:** 10.3389/fnsyn.2021.749001

**Published:** 2021-10-06

**Authors:** Benoit de Pins, Tiago Mendes, Albert Giralt, Jean-Antoine Girault

**Affiliations:** ^1^Institut du Fer à Moulin, Paris, France; ^2^Inserm UMR-S 1270, Paris, France; ^3^Faculté des Sciences et Ingénierie, Sorbonne Université, Paris, France; ^4^Departament de Biomedicina, Facultat de Medicina i Ciències de la Salut, Institut de Neurociències, Universitat de Barcelona, Barcelona, Spain; ^5^Institut d’Investigacions Biomèdiques August Pi i Sunyer (IDIBAPS), Barcelona, Spain; ^6^Centro de Investigación Biomédica en Red sobre Enfermedades Neurodegenerativas (CIBERNED), Madrid, Spain; ^7^Production and Validation Center of Advanced Therapies (Creatio), Faculty of Medicine and Health Science, University of Barcelona, Barcelona, Spain

**Keywords:** signal transduction, protein phosphorylation, glutamate receptors, learning and memory, neurodegenerative diseases

## Abstract

Pyk2 is a non-receptor tyrosine kinase highly enriched in forebrain neurons. Pyk2 is closely related to focal adhesion kinase (FAK), which plays an important role in sensing cell contacts with extracellular matrix and other extracellular signals controlling adhesion and survival. Pyk2 shares some of FAK’s characteristics including recruitment of Src-family kinases after autophosphorylation, scaffolding by interacting with multiple partners, and activation of downstream signaling pathways. Pyk2, however, has the unique property to respond to increases in intracellular free Ca^2+^, which triggers its autophosphorylation following stimulation of various receptors including glutamate NMDA receptors. Pyk2 is dephosphorylated by the striatal-enriched phosphatase (STEP) that is highly expressed in the same neuronal populations. Pyk2 localization in neurons is dynamic, and altered following stimulation, with post-synaptic and nuclear enrichment. As a signaling protein Pyk2 is involved in multiple pathways resulting in sometimes opposing functions depending on experimental models. Thus Pyk2 has a dual role on neurites and dendritic spines. With Src family kinases Pyk2 participates in postsynaptic regulations including of NMDA receptors and is necessary for specific types of synaptic plasticity and spatial memory tasks. The diverse functions of Pyk2 are also illustrated by its role in pathology. Pyk2 is activated following epileptic seizures or ischemia-reperfusion and may contribute to the consequences of these insults whereas Pyk2 deficit may contribute to the hippocampal phenotype of Huntington’s disease. Pyk2 gene, *PTK2B*, is associated with the risk for late-onset Alzheimer’s disease. Studies of underlying mechanisms indicate a complex contribution with involvement in amyloid toxicity and tauopathy, combined with possible functional deficits in neurons and contribution in microglia. A role of Pyk2 has also been proposed in stress-induced depression and cocaine addiction. Pyk2 is also important for the mobility of astrocytes and glioblastoma cells. The implication of Pyk2 in various pathological conditions supports its potential interest for therapeutic interventions. This is possible through molecules inhibiting its activity or increasing it through inhibition of STEP or other means, depending on a precise evaluation of the balance between positive and negative consequences of Pyk2 actions.

## Introduction

Focal adhesion kinases are cytoplasmic tyrosine kinases and scaffolding proteins involved in a wide variety of physiological and pathological processes including cell adhesion, cell migration, inflammatory responses, tumor invasiveness, neuronal development, and plasticity [reviews in Girault et al. (1999a); Schaller (2010); Walkiewicz et al. (2015); Zhu et al. (2018)]. This family of kinases encompasses two members, focal adhesion kinase (FAK, coded by the *Ptk2* gene in the mouse) and proline-rich tyrosine kinase 2 (Pyk2, *Ptk2b* gene). FAK and Pyk2 share about 45% amino acid sequence identity and 65% similarity (Avraham et al., 1995; Sasaki et al., 1995) but display very distinct expression patterns and some distinct properties. Whereas FAK is expressed in all tissues since early development, Pyk2 expression increases during the 3 weeks after birth in rat (Menegon et al., 1999). The full-length form of Pyk2 is highly expressed in the central nervous system (Avraham et al., 1995; Sasaki et al., 1995; Menegon et al., 1999). It is highly enriched in the pyramidal neurons of the hippocampus and to a lesser degree of the cerebral cortex, and also in the lateral septum, the thalamus, the amygdala, and the striatum (Menegon et al., 1999; Sheehan et al., 2003). Pyk2 is predominantly expressed in neurons but is also present in astrocytes (Cazaubon et al., 1997; Giralt et al., 2016). Pyk2 is highly expressed in monocytes and tissue-resident macrophages including osteoclasts (Duong et al., 1998) and microglial cells (Combs et al., 1999; Tian et al., 2000). In neurons, Pyk2 is regulated by neuronal activity and involved in synaptic plasticity (Girault et al., 1999a; Salter and Kalia, 2004). Mutant knockout mice without Pyk2 grow and breed well under laboratory conditions (Okigaki et al., 2003; Giralt et al., 2016) and the gross anatomy of their nervous system is normal (Giralt et al., 2016) showing that Pyk2 is not essential or can be functionally replaced possibly by FAK. However, recent insights have been provided by the study of the nervous system in Pyk2 mutant mice and its role in multiple pathological conditions, including neurodegenerative diseases. Here we review the properties and function of Pyk2 in the central nervous system with a focus on its role in synaptic function and dysfunction.

## Pyk2 a Multifunctional Protein Kinase With Scaffolding Properties

**FIGURE 1:**
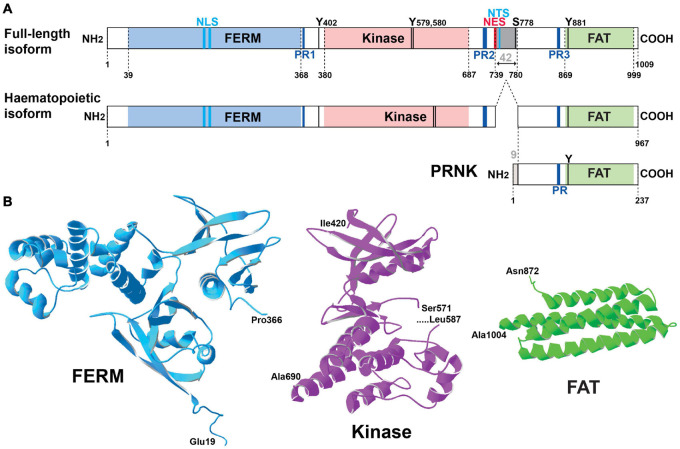
Pyk2 structure. **(A)** Schematic representation of Pyk2 protein isoforms. The limits of the structural domains are indicated with the first and last residue (numbering of mouse Pyk2): FERM, 4.1-ezrin-radixin-moesin, FAT, focal adhesion targeting. A Pyk2 protein isoform observed in cells of the hematopoietic lineage results from alternative splicing with the absence of an exon coding 42-amino acids (gray box). PRNK (Pyk2-related non-kinase) is a short isoform that derives from transcription initiation at an internal promoter and consists of the C-terminal region of Pyk2 with 9 additional residues at its N-terminus that are not found in Pyk2. Other important sites are indicated including the main characterized phosphorylation sites (T, threonine, Y, tyrosine), the proline-rich motifs (PR), the nuclear localization sequence (NLS), nuclear export sequence (NES), and nuclear targeting sequence (NTS). See text for references. **(B)** Three-dimensional structure of the main domains of Pyk2 for which the crystal structure has been determined. The structures were drawn with Deep View/Swiss Pdb viewer (http://www.expasy.org/spdbv/) for the FERM (pdb4eku), kinase (pdb4h1m), and FAT (pdb4gm3) domains.

Pyk2 is a 1009-amino acid, 110-kDa protein (Lev et al., 1995) that was independently discovered in several laboratories in different cellular models, and hence it is also known as fakB (Kanner et al., 1994), *cell adhesion kinase* β [CAKβ (Sasaki et al., 1995)], *related adhesion focal tyrosine kinase* (RAFTK) (Avraham et al., 1995), and *calcium-dependent protein-tyrosine kinase* (CADTK) (Yu et al., 1996). Pyk2 encompasses several well-defined structural domains: an N-terminal FERM (4.1/ezrin/radixin/moesin) domain (Chishti et al., 1998; Girault et al., 1998, 1999b), a central tyrosine kinase domain, and a C-terminal focal adhesion targeting domain (FAT) (Hildebrand et al., 1993; Figure 1A). The short linker of 43 residues in mouse sequence between the FERM and kinase domains contains a proline-rich motif (PR1) and the autophosphorylation site, Tyr402. The longer linker of 171 residues in the mouse sequence between the kinase and FAT domain (referred below to as kinase-FAT linker, KFL) includes 2 additional proline-rich sequences (PR2 and PR3). The KFL region encompasses the most divergent sequence between FAK and Pyk2. The structure of the FERM (PDB ID 4EKU), kinase (Han et al., 2009), and FAT (Lulo et al., 2009) domains of Pyk2 has been determined by X-ray crystallography (Figure 1B) and is, as expected based on high sequence identity, very similar to that of corresponding domains in FAK. In contrast, the linkers appear to be disordered flexible regions important for Pyk2 interactions and regulation. In addition to the main full-length form of Pyk2, two protein isoforms encoded by the same gene have been described (Figure 1A). The first isoform is a splice variant expressed in hematopoietic cells, which lacks an exon encoding 42 amino acids (739–780 in rat) in KFL, between PR2 and PR3 (Dikic et al., 1998; Xiong et al., 1998). The second Pyk2 isoform, presumably transcribed from an internal initiation site, lacks the FERM and kinase domains and is referred to as Pyk2-related non-kinase (PRNK) (Xiong et al., 1998) or CRNK (Li et al., 1999). It consists of the 228 C-terminal residues of Pyk2 fused to nine unique N-terminal amino acids (Xiong et al., 1998; Figure 1A). PRNK is expressed in several tissues including the brain and may act as an endogenous regulator of Pyk2 activity (Xiong et al., 1998). Numerous proteins interacting with Pyk2 have been identified, including many also interacting with FAK. For some of these partners good evidence supports a direct interaction with Pyk2 (Table 1), whereas for others the interaction is likely to be indirect or insufficiently characterized (Table 2). Many of the direct interactions are mediated by partners’ Src-homology 3 (SH3) domain binding to Pyk2 PR motifs, while some others depend on Src-homology 2 (SH2) domain binding to specific phosphorylated tyrosine residues in Pyk2 sequence and are triggered by its activation (Table 1). Therefore Pyk2 is considered to be a scaffolding protein with multiple protein-protein interactions.

**TABLE 1:** Protein partners very likely or shown to interact directly with Pyk2.

Partner	Partner binding region	Binding site in Pyk2	Comments	References
ArgBP2	SH3A	PR2 and/or PR3	PC12, HEK293T; co-IP, GST pull-down; complex with Pyk2, Cbl	Haglund et al. (2004)
ASAP1 / DEF-1 / PIP2-dependent ARF1 GAP	SH3	PR2 and/or PR3	Rat brain, HEK293, PC12; Pyk2 P717A or P859A reduces ASAP1 phosphorylation	Kruljac-Letunic et al. (2003)
ASAP2 / PAG3 / PAP	SH3?			Andreev et al. (1999)
BCAR1 / p130cas	SH3	PR2 and/or PR3		Astier et al. (1997); Manie et al. (1997); Ohba et al. (1998); Anfosso et al. (2001); Takahashi et al. (2003)
Related to p130Cas: embryonal Fyn-associated substrate (EFS)/ SIN	SH3	670–792	Interacts more with FAK than Pyk2	Ohba et al. (1998)
Related to p130Cas: enhancer of filamentation 1 (hEF1 / NEDD9 / CasL)	SH3	PR2 and/or PR3	Similar to p130-Cas	Manie et al. (1997)
Calmodulin	With Ca^2+^	FERM, kinase, KFL	Multiple, see text	Kohno et al. (2008); Xie et al. (2008); Vomaske et al. (2010)
Disks large homolog 3 (DLG3) / Synapse-associated protein 102 (SAP102)	SH3	PR2 and/or PR3		Seabold et al. (2003)
Disks large homolog 4 (DLG4) / PSD-95	SH3	PR2 and/or PR3	Requirement of the proline-rich region of Pyk2 for binding to PSD-95 and SAP102	Seabold et al. (2003)
FGFR-2, -3c	C-ter	Kinase domain?		Meyer et al. (2004)
Gelsolin	C-ter	PR3	HEK293, co-IP formation of osteoclastic actin rings	Wang et al. (2003)
Graf1C (RhoA GTPase-activating protein)	SH3	PR3 > PR2	Pyk2 inhibits Graf1c (requires kinase activity)	Lee et al. (2019)
Grb2	SH2	pTyr881	Grb2/Ras/Raf/MEK activates ERK1/2 after short treatment of GT1-7 cells with GnRH	Li et al. (1996); Ganju et al. (1997); Blaukat et al. (1999); Bruzzaniti et al. (2009); Okitsu-Sakurayama et al. (2018)
Grm1, metabotropic glutamate receptor 1, mGluR1	2^nd^ intracellular loop, C-ter tail		Pyk2 displaces Gαq/11 from the receptor and activates ERK	Nicodemo et al. (2010)
Grm5, metabotropic glutamate receptor 5, mGluR5	2^nd^ intracellular loop, C-ter tail		Similar effects as Grm1?	Nicodemo et al. (2010)
Integrin beta-3 / CD61 / GP3a	C-ter 17 aa	N-terminal	Pyk2 downstream of β3; *in vitro* binding	Pfaff and Jurdic (2001)
Megakaryocyte-associated tyrosine-protein kinase / CsK homologous kinase (CHK) / MATK	SH2	pY402	CHK inhibits Pyk2 phosphorylation; co-IP	McShan et al. (2002)
Membrane-associated phosphatidylinositol transfer protein 1, 2, 3 / PITPnm 1, 2, 3 / NIR-2, -3, -1/RdgB-homologs	C-terminal	FERM	Yeast two-hybrid assay; binding, co-IP, Pyk2 substrates	Lev et al. (1999)
Methyl-CpG-binding domain protein 2 (MBD2)	MBD domain	FAT?	Decreases MBD2 association with HDAC1 and methyl CpG site; binding, co-IP	Luo et al. (2009)
Nephrocystin-1, -4	N-ter, SH3	PR3	Complex with p130Cas, tensin	Benzing et al. (2001); Mollet et al. (2005)
Paxillin	LD domains	FAT		Hiregowdara et al. (1997); Matsuya et al. (1998); Zheng et al. (1998); Ivankovic-Dikic et al. (2000); Anfosso et al. (2001); Vanarotti et al. (2014)
Leupaxin (LIM domains, LD, similar to paxillin)	LD	FAT	Complex with PTP-PEST; binding, co-IP	Lipsky et al. (1998); Sahu et al. (2007); Vanarotti et al. (2016)
HIC-5 (LIM domains, LD, similar to paxillin)	LD	FAT	Binding, co-IP	Matsuya et al. (1998); Wang et al. (2002)
PLC-γ1, -γ2	C-ter SH2 and SH3	pTyr and/or PR	Complex with β3 integrin, PLC-γ, phosphatidylinositol 3-kinase	Nakamura I. et al. (2001)
Protein kinase MAP4K4 (mitogen-activated protein kinase 4)	C-terminal citron homology domain (CNH)	FERM	Role in glioma cell migration	Loftus et al. (2013)
PTK Abl	SH2	pTyr881	Complex with pl90 RhoGAP (pi90), RasGAP, c-Abl, pl30cas, paxillin.	Zrihan-Licht et al. (2004)
PTK Fyn / p59-Fyn	SH2	pTyr402		Ganju et al. (1997); Qian et al. (1997); Marie-Cardine et al. (1999); Katagiri et al. (2000); Li and Gotz (2018)
PTK Lck / p56-Lck	SH2		Co-IP	Ganju et al. (1997); Qian et al. (1997)
PTK Lyn / p56-Lyn	SH2?			Yamasaki et al. (2001)
PTK Src / p60-Src	SH2, SH3?	pTyr402, PR1?		Dikic et al. (1996); Duong et al. (1998); Kumar et al. (1999); Keely et al. (2000); Sanjay et al. (2001); Faccio et al. (2003); Lakkakorpi et al. (2003); Bruzzaniti et al. (2009); Hum et al. (2014)
PTK Yes (c-Yes / p61-Yes)	SH2?		Pancreatic acinar cells	Sancho et al. (2012)
PTK ZAP-70	SH2?			Katagiri et al. (2000)
PTP non-receptor type 11 (SHPTP2 / SHP2)	Not the SH2 domain	pTyr402	Pyk2 is a substrate for SHPTP2 in response to dexamethasone and IL-6	Chauhan et al. (2000)
PTP non-receptor type 5 / Striatum-enriched protein-tyrosine phosphatase (PTPN5 / STEP)	PR2 and KIM domains of STEP61	671–694	Dephosphorylates pY402	Xu et al. (2012)
Proto-oncogene vav (VAV1)	SH3 (605–662) and SH3 (786–844)	PR?	Complex with ZAP-70	Katagiri et al. (2000)
FAK family kinase–interacting protein of 200 kD (FIP200) / RB1-inducible coiled-coil protein 1	C-ter	Kinase domain	Yeast 2-hybrid, co-IP, FIP200 inhibits Pyk2	Ueda et al. (2000)
RNA-binding protein EWS (EWSR1)	N-ter	656–797	Co-IP, pull-down	Felsch et al. (1999)
SAP90/PSD-95-associated protein 3 (SAPAP3) / Disks large-associated protein 3 (DAP3)	C-ter (436–977)		Yeast 2-hybrid (FAK), GST-pulldown	Bongiorno-Borbone et al. (2005)
Src kinase-associated phosphoprotein 2 (SKAP2) / Pyk2/RAFTK-associated protein (PRAP)	SH3	C-ter region	Yeast 2-hybrid, co-IP, may associate Pyk2 and α-synuclein	Takahashi et al. (2003)
Suppressor of cytokine signaling 2 (SOCS2); SOCS3	SH2	pY402	SOCS2 induces proteasome-mediated degradation pPyk2 via ubiquitination	Zhang et al. (2008); Lee et al. (2010)

*Co-IP, co-immunoprecipitation; GST, glutathione-S transferase; KIM, kinase interaction motif; LD, leucine-aspartic acid motif; LIM, acronym of the three genes in which the domain was first identified: LIN-11, Isl-1 and MEC-3; PR, proline-rich motif; PTK, protein tyrosine kinase; PTP, protein tyrosine phosphatase; SH2, SH3, Src-homology 2, 3. Question marks indicate interacting domains that are not directly identified but very likely involved based on similarity with other characterized interactions.*

**TABLE 2:** Protein partners possibly interacting indirectly with Pyk2.

Partner	Comments	Reference
α1-actin, α2-actin, β-actin	pUS28-expressing U373 or RSMC; IP, tandem MS	Vomaske et al. (2010)
Abl-interactor-2	pUS28-expressing U373; IP, tandem MS; low levels	Vomaske et al. (2010)
AF-17	pUS28-expressing RSMC; IP, tandem MS	Vomaske et al. (2010)
Alpha-1,3/1,6-mannosyltransferase / ALG2	Co-precipitation of FAK, Pyk2	Schmidt et al. (2003)
Annexin VI / annexin A6	COS-1, Rat-1; GST pull-down, co-IP; complex with Fyn, Pyk2, p120GAP	Chow et al. (2000)
ARF GTPase-activating protein GIT1 / CAT-1		Ko et al. (2003)
BiP	pUS28-expressing U373; IP, tandem MS	Vomaske et al. (2010)
Copine-5	pUS28-expressing U373; IP, tandem MS; low levels	Vomaske et al. (2010)
Dynamin	Possibly mediated by Grb2	Bruzzaniti et al. (2009)
E3 ubiquitin-protein ligase / Cbl /RING finger protein 56	Osteoclasts, PC12, HEK293; co-IP; contributes to Pyk2 degradation. Involves Cbl C-terminal region	Sanjay et al. (2001); Schmidt et al. (2003); Haglund et al. (2004); Fan et al. (2014)
E3 ubiquitin-protein ligase CBLB / Cbl-b	MGC803; co-IP; contributes to Pyk2 degradation	Fan et al. (2014)
E3 ubiquitin-protein ligase NEDD4	Pyk2 and FAK	Verma et al. (2017)
EGFR / ErbB-1	Pyk2 and FAK, neurite outgrowth regulation	Ivankovic-Dikic et al. (2000); Keely et al. (2000)
ErbB-2 / HER2 / CD340	Pyk2 mediates activation of MAP kinase by neuregulins. Involves Pyk2 pY402	Zrihan-Licht et al. (2000)
ErbB-3 / HER3	PYK2 regulates the degradation of HER3	van der Horst et al. (2005); Verma et al. (2017)
F-actin capping proteins-a1, -a2, and –b	pUS28-expressing RSMC; IP, tandem MS; low levels	Vomaske et al. (2010)
GPIb-β; GPVI	Human platelets; co-IP; indirect	Arthur et al. (2011)
Grin2a, glutamate receptor ionotropic NMDA 2A, GluN2A, NR2A	See text	Liu et al. (2001); Seabold et al. (2003)
Grin2b, glutamate receptor ionotropic NMDA 2B, GluN2B, NR2B	See text	Seabold et al. (2003)
G-protein subunit alpha-13 (Gα13)	Pyk2 downstream of Gα13; Co-IP	Shi et al. (2000)
G-protein subunit beta-5 (Gβ5)	pUS28-expressing RSMC; IP, tandem MS; low levels	Vomaske et al. (2010)
Hsc70	pUS28-expressing U373; IP, tandem MS	Vomaske et al. (2010)
Integrator complex subunit 6	pUS28-expressing U373; IP, tandem MS	Vomaske et al. (2010)
Integrin beta-1 / CD29 / MDF2	Possibly through PI3K	Melikova et al. (2004)
Integrin beta-2 / CD18 / MF17	Pyk2 downstream of β2 not associated	Yan and Novak (1999); Miura et al. (2000); Evangelista et al. (2007)
Interleukin-7 receptor alpha (IL-7R-alpha)	Increased by IL-7	Benbernou et al. (2000)
Janus kinase 1 (JAK1)	Increased by IL-7	Benbernou et al. (2000)
Janus kinase 2 (JAK2)	Pyk2 associated with and downstream of Jak2; co-IP	Takaoka et al. (1999); Frank et al. (2002)
Janus kinase 3 (JAK3)	Increased by IL-2; co-IP	Miyazaki et al. (1998)
KSR-2	pUS28-expressing RSMC; IP, tandem MS low levels	Vomaske et al. (2010)
Microtubule associated protein Tau	Tau Pyk2-substrate	Dourlen et al. (2017); Li and Gotz (2018)
Mortalin	pUS28-expressing U373; IP, tandem MS	Vomaske et al. (2010)
Myosin light chain-6	pUS28-expressing RSMC; IP, tandem MS low levels	Vomaske et al. (2010)
Myosin-9	pUS28-expressing U373; IP, tandem MS	Vomaske et al. (2010)
N-acetyltransferase-10	pUS28-expressing U373; IP, tandem MS	Vomaske et al. (2010)
Nectin-3	pUS28-expressing U373; IP, tandem MS	Vomaske et al. (2010)
NFkB p105	pUS28-expressing RSMC; IP, tandem MS low levels	Vomaske et al. (2010)
NIM1 kinase	pUS28-expressing U373; IP, tandem MS	Vomaske et al. (2010)
Phosphatidylinositol 3-kinase regulatory subunit alpha (PIK3R1 / PI3K-p85)	Complex with integrin beta-1 / CD29 / MDF2 Co-IP	Melikova et al. (2004)
Potassium voltage-gated channel subfamily A member 2 (KCNA2 / Kv1.2 / NGK1)	Pyk2 phosphorylates and may inhibit Kv1.2; co-IP	Byron and Lucchesi (2002)
Programmed cell death 6-interacting protein (PDCD6-interacting protein) / ALG-2-interacting protein 1 (AIP1 /Alix)	Complex with SETA/CIN85/Ruk and AIP1; HEK293 cells; co-IP	Schmidt et al. (2003)
Protein kinase C, PKC-alpha	Complex with β1-integrin and PI3K; myeloma cells co-IP	Podar et al. (2002)
Protein kinase C, PKC-delta	Pancreatic acinar cells, co-IP	Wrenn (2001)
PTK SYK	Complexes with Syk, SHP1, and Grb2, with β2 integrin	Ganju et al. (2000); Miura et al. (2000)
PTP non-receptor type 11 (SHPTP2 / SHP2)	Pyk2 substrate of SHPTP2; interaction involves pY402 but not SHP2 SH2	Chauhan et al. (2000)
PTP non-receptor type 12 (PTP-PEST / PTPG1)	PTP-PEST dephosphorylates pY402, pY579 and pY580	Lyons et al. (2001)
PTP non-receptor type 6 (SHPTP1 / SHP1)	SHPTP1 prevents Pyk2 phosphorylation; co-IP	Kumar et al. (1999); Ganju et al. (2000)
Ras GTPase-activating protein 1 (RasGAP / p120GAP)	Complex with pl90 RhoGAP (pi90), RasGAP, c-Abl, pl30cas, paxillin; C2 domain of RasGAP	Chow et al. (2000); Zrihan-Licht et al. (2000)
Rho GTPase-activating protein 5 (p190RhoGAP)	Complex with pl90 RhoGAP (pi90), RasGAP, c-Abl, pl30cas, paxillin	Zrihan-Licht et al. (2000)
Rhophilin-2	pUS28-expressing RSMC; IP, tandem MS; low levels	Vomaske et al. (2010)
RTEL-1	pUS28-expressing RSMC; IP, tandem MS	Vomaske et al. (2010)
SH3 domain-containing kinase-binding protein 1 (CD2BP3 / SETA / RUK / CIN85)	Complex with SETA/CIN85/Ruk and AIP1; HEK293 cells; co-IP	Schmidt et al. (2003)
TCF-4	pUS28-expressing U373; IP, tandem MS	Vomaske et al. (2010)
PTP (TFG) Trk-fused gene	pUS28-expressing RSMC; IP, tandem MS; low levels	Vomaske et al. (2010)
TNF receptor-associated factor 4 (Traf4)	Complex with Hic-5	Wu et al. (2005); Arthur et al. (2011)
Trio	pUS28-expressing RSMC; IP, tandem MS; low levels	Vomaske et al. (2010)
Tropomodulin-3	pUS28-expressing RSMC; IP, tandem MS; low levels	Vomaske et al. (2010)
Tropomyosin-a1, a3, a4, -b	pUS28-expressing RSMC; IP, tandem MS; low levels	Vomaske et al. (2010)
Tubulin-b-2C	pUS28-expressing RSMC; IP, tandem MS; low levels	Vomaske et al. (2010)
USP6 oncogene	pUS28-expressing U373; IP, tandem MS	Vomaske et al. (2010)
VEGFR-1 / FLT-1		Podar et al. (2002)
Vimentin	pUS28-expressing RSMC; IP, tandem MS; low levels	Vomaske et al. (2010)

*IP, immunoprecipitation; MS, mass spectrometry; PTK, protein tyrosine kinase; PTP, protein tyrosine phosphatase; RSMC, rectal smooth muscle cells.*

## Pyk2 Autophosphorylation and Regulation of by Ca^2+^

### Autophosphorylation of Pyk2 and Interactions With Src-Family Kinases

**FIGURE 2:**
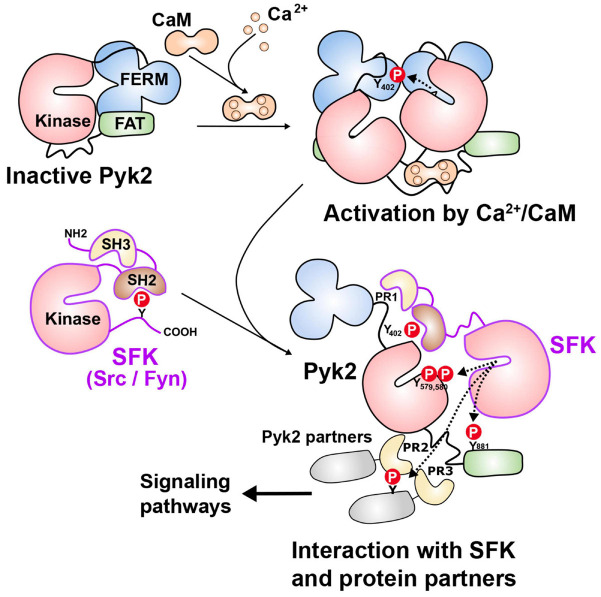
Pyk2 mechanisms of activation. In the absence of stimulus, Pyk2 is thought to be in a closed conformation due to possible intramolecular interactions between domains (top left). Activation can result from the binding of Ca^2+^/calmodulin (Ca^2+^/CaM) for which several potential sites have been identified, including in the FERM domain, the kinase domain, and the kinase-FAT linker (as shown here for simplicity). The binding of Ca^2+^/calmodulin is thought to facilitate dimerization or clustering of Pyk2 allowing intermolecular autophosphorylation of Tyr402 (Y_402_). Clustering can also result from interaction with partners including PSD-95, which can themselves dimerize or cluster in response to Ca^2+^/calmodulin (not shown). Autophosphorylation of Y_402_ provides a binding site for the SH2 domain of Src family kinases (SFKs especially Src and Fyn). Through their SH3 domain, SFKs can also bind to the proline-rich motif (PR1) located close to Y_402_. SFKs can then phosphorylate several tyrosine residues in Pyk2 including Y_579_ and Y_580_ in its activation loop, increasing its catalytic activity, and Y_881_ in the FAT domain, which can recruit additional partners with SH2 domains. SFKs, and possibly Pyk2 can phosphorylate associated proteins, including those interacting with PR2 and PR3 motifs through their SH3 domains. This can result in the formation of multiprotein complexes and the activation of various signaling pathways. Alternatively, activated SFKs can bypass the Ca^2+^/calmodulin-dependent step by directly phosphorylating Y_402_. In either case SFK and Pyk2 interactions provides a positive feedforward activation loop. See text for references.

The most striking structural feature of Pyk2 is the presence of a central tyrosine kinase domain and many studies explored its regulation. Activation of protein tyrosine phosphorylation by depolarization and neurotransmitters was reported in neurons in culture and hippocampal slices more than 25 years ago (Siciliano et al., 1994). Two of the main proteins whose phosphorylation was increased were then identified as FAK and Pyk2 (Siciliano et al., 1996). Pyk2 was initially characterized as a Ca^2+^-activated protein tyrosine kinase (Lev et al., 1995) and also termed calcium-dependent protein-tyrosine kinase (Yu et al., 1996). A remarkable property of FAK and Pyk2 is the existence of an autophosphorylation site in the FERM-kinase linker whose phosphorylation is an early step in the activation of these two proteins. Due to the sequence similarities between the two enzymes, the current model of Pyk2 activation is in part based on results obtained with FAK which are presumed to be also valid for Pyk2. In Pyk2 the autophosphorylated tyrosine is Tyr402, which promotes its interaction with the SH2 domain of Src-family kinases (SFKs) (Li et al., 1999). The SFKs reported to interact with Pyk2 include Src (Dikic et al., 1996; Duong et al., 1998; Kumar et al., 1999; Keely et al., 2000; Sanjay et al., 2001; Faccio et al., 2003; Lakkakorpi et al., 2003; Bruzzaniti et al., 2009; Hum et al., 2014) and Fyn (Ganju et al., 1997; Qian et al., 1997; Marie-Cardine et al., 1999; Katagiri et al., 2000; Li and Gotz, 2018), which are both widely expressed in neurons, as well as Lck (Ganju et al., 1997; Qian et al., 1997), Lyn (Yamasaki et al., 2001), and Yes (Sancho et al., 2012). Because the SH2 and SH3 domains involved in the interactions with FAK and Pyk2 are conserved among SFKs, we refer to them below as a generic family, although the detailed interactions have not been documented in all cases. SFKs are also likely to bind to the nearby PR1 motif of Pyk2 through their SH3 domain as it was shown for FAK (Thomas et al., 1998). The binding of SFKs to Pyk2 is expected to activate these enzymes by competing with the intramolecular interactions of SFK including binding of their SH2 domain to an inhibitory phosphotyrosine in their carboxy-terminal region and of their SH3 domain to a motif between the kinase and SH2 domain (Roskoski, 2015; Figure 2). SFKs can phosphorylate several residues in Pyk2, including Tyr579 and Tyr580 in its activation loop, whose phosphorylation is expected to enhance its kinase activity (Li et al., 1999). Pyk2 phosphorylation at Tyr881 can recruit the adaptor Grb2 (Felsch et al., 1998) and the tyrosine kinase Abl (Zrihan-Licht et al., 2004). The phosphorylation of Pyk2 on several tyrosine residues combined with its interaction with multiple partners triggers various signaling pathways, in part depending on the cell type. Phosphorylation of Pyk2 at Tyr402 can result from autophosphorylation and recruit SFKs as outlined above, but SFKs can phosphorylate other residues in Pyk2 independently of Tyr402 (Li et al., 1999) and also directly phosphorylate Tyr402 (Higa-Nakamine et al., 2020; Das et al., 2021). This phosphorylation by SFKs may be an indispensable primer for further autophosphorylation in some circumstances (Zhao et al., 2016). Thus there is a clear positive feedforward loop between Pyk2 and SFKs, involving their reciprocal activating interactions. This may favor their strong activation in responses to stimuli, but makes the causal relationship between these events difficult to analyze. Accordingly, in the hippocampus, Fyn plays an important role in Pyk2 tyrosine phosphorylation including at Tyr402, which is markedly decreased in basal conditions and response to stimuli in Fyn KO mice (Corvol et al., 2005).

### The Role of Ca^2+^ in Pyk2 Activation

The mechanism by which increased intracellular Ca^2+^ activates Pyk2 is not fully understood but several observations provide some insights. As shown for FAK (Toutant et al., 2002; Brami-Cherrier et al., 2014) Pyk2 is autophosphorylated by an intermolecular reaction (Park et al., 2004). In inactive Pyk2, the FERM domain is likely to inhibit the kinase domain through an intramolecular interaction as proposed for FAK (Lietha et al., 2007). This interaction may also be involved in oligomer formation which promotes intermolecular autophosphorylation, whereas an excess of free FERM domain decreases autophosphorylation (Riggs et al., 2011). In the case of FAK, the existence of several intramolecular, and potentially intermolecular interactions have been identified (Lietha et al., 2007; Brami-Cherrier et al., 2014). The FAK FERM domain can dimerize through a tryptophan residue (Trp-266) that is conserved in Pyk2 (Trp-273), providing a basis for an intermolecular interaction favoring *trans-*autophosphorylation when the intramolecular inhibition of the kinase by the FERM domain is relieved by other factors (Brami-Cherrier et al., 2014). Whether the same scheme applies to Pyk2 and how Ca^2+^ contributes to relieving the inhibition exerted by the FERM domain and/or facilitates dimerization remains to be determined.

Ca^2+^-induced autophosphorylation is prevented by calmodulin (CaM) antagonists and direct action of Ca^2+^/CaM on Pyk2 has been proposed with at least two potential binding sites, one in the FERM domain (including L_176_-Q_177_) (Kohno et al., 2008) and another in the kinase domain (L_514_ERNKNSLKVPTLV) (Xie et al., 2008). The binding of Ca^2+^/CaM to the FERM domain was reported to facilitate Pyk2 dimerization, providing a potential mechanism for its autophosphorylation (Kohno et al., 2008). However, the molecular mechanisms initiated by Ca^2+^/CaM binding at these sites leading to intermolecular phosphorylation are not known and the existence of an additional site is plausible (Figure 2). Because Pyk2 interacts with multiple protein partners, Ca^2+^ can also potentially act on these partners to facilitate Pyk2 intermolecular autophosphorylation through its dimerization or clustering. For example, the SH3 domain of PSD-95 binds the Pyk2 PR3 motif (Seabold et al., 2003) and Ca^2+^/CaM promotes the dimerization of PSD-95 and thereby activates Pyk2 (Bartos et al., 2010). Moreover, CaM is reported to activate Src in the presence or even in the absence of Ca^2+^ (Stateva et al., 2015).

In addition to a direct implication of Ca^2+^/CaM, many reports have shown the importance of serine/threonine phosphorylation in Pyk2 regulation. Indeed, Pyk2 activation can be triggered by stimulating PKC and its activation is blocked by PKC inhibitors in various preparations (Siciliano et al., 1994; Lev et al., 1995; Siciliano et al., 1996; Hiregowdara et al., 1997). How PKC activates Pyk2 autophosphorylation is not known and may result from indirect effects, such as activation of Src (Cao et al., 2007). In some cell types, Pyk2 autophosphorylation induced by depolarization but not by G protein-coupled receptor stimulation is prevented by Ca^2+^/CaM-dependent protein kinase inhibitors (Zwick et al., 1999; Ginnan and Singer, 2002), indicating that this group of kinases is also capable to contribute to Pyk2 activation, directly or indirectly. Finally, inhibition of calcineurin (also known as PP2B or PPP3), a Ca^2+^- and Ca^2+^/CaM-activated serine/threonine phosphatase, also prevents depolarization-induced Pyk2 autophosphorylation (Faure et al., 2007). Although calcineurin dephosphorylates pSer778 in Pyk2, this residue is important for nuclear export of Pyk2 but not for Tyr402 phosphorylation (Faure et al., 2013) suggesting the existence of other important sites for Pyk2 activation, on either Pyk2 or other proteins involved in its regulation, including STEP (see Section 4 below). While the mechanistic aspects of Pyk2 regulation by Ca^2+^ are still imperfectly understood, it is striking that it is a point of convergence of multiple Ca^2+^-activated pathways, indicating that Pyk2 behaves as a broad sensor of Ca^2+^ regulation. Other signals activating Pyk2 have been reported without full elucidation of their mechanisms of action, including hyperosmotic shock (Tokiwa et al., 1996; Derkinderen et al., 1998), acidic pH (Li et al., 2004), and SUMOylation (Uzoma et al., 2018), but their relevance in neurons is not known.

## Negative Regulation of Pyk2 by Tyrosine Phosphatases and Protocadherins

Termination of Pyk2 activation results from its dephosphorylation by protein tyrosine phosphatases (PTPs). Several PTPs active on Pyk2 have been identified in non-neuronal cells including SHP1 (Kumar et al., 1999), SHP2 (Tang et al., 2000; Halfter et al., 2005), and PTP-PEST (a.k.a. PTPN12) (Davidson and Veillette, 2001; Lyons et al., 2001). In neurons, PTPα decreases Pyk2 phosphorylation either directly or by regulating SFKs (Le et al., 2006), but the phosphatase which appears to play the most prominent role is STEP (also known as protein tyrosine phosphatase non-receptor type 5, PTPN5), which is specifically enriched in the neuronal populations highly expressing Pyk2 (Fitzpatrick and Lombroso, 2011; Lombroso et al., 2016). STEP binds to Pyk2 and catalyzes the dephosphorylation of Tyr402 (Xu et al., 2012). Phosphorylation of Pyk2 Tyr402 is increased in the hippocampus of STEP KO mice (Venkitaramani et al., 2011). STEP is negatively regulated by phosphorylation by cAMP-dependent protein kinase on a key serine residue, and activated by dephosphorylation of this residue by protein phosphatase 1 (PP1 or PPP1) which is itself activated by calcineurin (Goebel-Goody et al., 2012). These phosphorylation-dependent regulations provide an interesting possibility of crosstalk between the cAMP pathway, calcineurin, and Pyk2/SFKs. It could also contribute to the effects of calcineurin inhibitors on the autophosphorylation of Pyk2 (Faure et al., 2007). In addition to tyrosine phosphatases, endogenous inhibitors of Pyk2 have been identified specifically in the central nervous system. Among them, α- and γ-protocadherins were shown to interact with and negatively regulate Pyk2 thereby modulating synaptic connectivity (Suo et al., 2012), neuronal migration (Fan et al., 2018), and cell survival (Chen et al., 2009a). Thus the two best characterized negative regulators of Pyk2 are STEP, a phosphatase regulated by multiple signaling pathways and protocadherins.

## Dynamics of Pyk2 Localization in Neurons

### Pyk2 Accumulation in Post-synaptic Densities

**FIGURE 3:**
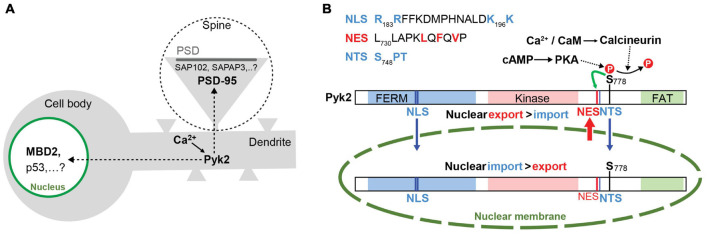
Dynamic changes in Pyk2 localization in neurons. **(A)** In the absence of stimulus Pyk2 is mostly localized in the cytoplasm. In response to Ca^2+^ influx (e.g., following depolarization or NMDAR stimulation), Pyk2 accumulates in the nucleus where it can interact with partners identified in non-neuronal cells (MBD2, p53) and in postsynaptic densities (PSDs) in dendritic spines (one is magnified in the dotted circle), where it can bind to PSD-95, SAP102 or SAPAP3 (see text for references and Figure 4). **(B)** Summary of the cytonuclear fluxes of Pyk2. Identified motifs in Pyk2 sequence known to be a nuclear export sequence (NES), a nuclear targeting sequence (NTS), and a nuclear localization signal (NLS). The localization of Pyk2 depends on the difference between its nuclear import and export rates. The nuclear import results from the interaction of NLS and NTS with specific carriers for transport through the nuclear pores (symbolized as an interruption in the nuclear membrane). The nuclear export depends on the activity of the NES motif that is potentiated by phosphorylation of Ser778 (**S_778_**). Dephosphorylation of S_778_ by calcineurin in response to Ca^2+^ increase impairs the efficacy of NES to promote nuclear export and results in an increase of Pyk2 concentration in the nucleus. See text for references after Faure et al. (2013).

Pyk2 is a cytoplasmic protein that can accumulate at contact or adhesion sites in specific cell types and conditions including podosomes and invadopodia [see (Eleniste and Bruzzaniti, 2012; Genna and Gil-Henn, 2018) for reviews]. In neurons, Pyk2 is rather diffusely distributed but its localization is altered after stimulation (Figure 3A). Following depolarization or activation of glutamate NMDA receptor (NMDAR) Pyk2 is rapidly clustered at post-synaptic sites in spines and this clustering is prevented in the presence of the PSD-95 SH3 domain (Bartos et al., 2010). In addition to PSD-95, postsynaptic enrichment of Pyk2 can also be facilitated by its association with other PSD proteins including the SH3 domain of SAP102 (Seabold et al., 2003) and SAPAP3 (Bongiorno-Borbone et al., 2005). As we shall see below, in the PSD Pyk2 can modify neuronal excitability by inducing the phosphorylation of ion channels (Lev et al., 1995; Felsch et al., 1998; Huang et al., 2001).

### Pyk2 Cytonuclear Shuttling and Nuclear Accumulation

In addition to its stimulation-induced accumulation in PSDs, Pyk2 also shuttles between the cell nucleus and the cytoplasm (Figure 3B). In various cell lines, Pyk2 accumulates in the nucleus following mutation of Pro-859 to Ala in the PR3 motif or treatment with nuclear export inhibitor leptomycin B (Aoto et al., 2002). In transfected cells stimulation of gonadotropin-releasing hormone (GnRH) receptor increases Pyk2 phosphorylation and induces its nuclear accumulation (Farshori et al., 2003) and, during early development, Pyk2 is transiently localized to the nucleus (Meng et al., 2006). In hippocampal slices, neurons in culture, and PC12 cells, depolarization induces a Ca^2+^/CaM- and calcineurin-dependent nuclear translocation of Pyk2 (Faure et al., 2007). Interestingly, high frequency stimulation of Schaffer collaterals in hippocampal slices induces nuclear accumulation of Pyk2 in CA1 pyramidal neurons (Faure et al., 2007), suggesting it occurs in conditions known to trigger synaptic long-term potentiation (LTP, see below section “Pyk2 in Long Term Potentiation”). Pyk2 contains several nuclear import and export signals (see Figures 1, 3B), including a nuclear export motif regulated by phosphorylation at Ser778, a substrate of cAMP-dependent protein kinase and calcineurin (Faure et al., 2013). Upon depolarization, calcineurin dephosphorylates Ser778 leading to the inactivation of the nuclear export motif and the accumulation of Pyk2 in the nucleus (Faure et al., 2013). The function of Pyk2 in the nucleus remains unclear. Nuclear Pyk2 has the potential to downregulate p53 by increasing p53 ubiquitination through its FERM domain thereby favoring cell survival (Lim et al., 2008a; Lim et al., 2010). In addition, it has been shown in keratinocytes and osteocytes that FAK and Pyk2 interact with methyl-CpG-binding domain protein-2, MBD2 (Schindler et al., 2007; Hum et al., 2014). MBD2 is a methylated-DNA binding protein that recruits histone deacetylase to the DNA (Luo et al., 2009). Interaction with Pyk2 prevents MBD2 association with both HDAC1 and methyl-DNA and thereby increases gene expression (Luo et al., 2009). Whether this regulation also takes place in neurons and whether and how it affects transcription in response to signals that activate Pyk2 is not known. In particular, it will be of interest to determine whether the nuclear role of Pyk2 is targeted to specific genes or is a general modulation that alters responsiveness to more specific pathways. Because it provides a potential link between neuronal activity and transcriptional responses the role of Pyk2 in the nucleus in neurons deserves further investigation including in the context of synaptic plasticity.

## Pyk2 in Synapses

### Pyk2 in Neurite and Spine Formation and Maintenance

In non-neuronal cells such as osteoclasts, Pyk2 is implicated in the regulation of actin-cytoskeleton downstream of integrins through its interaction with proteins like the Crk-associated substrate p130Cas (also known as breast cancer anti-estrogen resistance protein 1, BCAR1) and paxillin, in a manner similar to FAK [review in Kong et al. (2020)]. Both FAK and Pyk2 are present in neurite growth cones (Menegon et al., 1999). The expression of the C-terminal domain of Pyk2 or FAK blocks neurite outgrowth in PC12 and SH-SY5Y cells indicating the possible implication of the FAK family in this process (Ivankovic-Dikic et al., 2000). Supporting this hypothesis, Pyk2 forms a signaling complex with Cbl, an E3 ubiquitin-protein ligase, and Arg kinase-binding protein 2 (ArgBP2), an adaptor protein, which may regulate actin to form lamellipodia in growth cones of differentiating PC12 cells following growth factor stimulation (Haglund et al., 2004). Pyk2 is also activated and associates with paxillin and bundled actin at neurite initiation sites in PC12 cells in response to nerve growth factor (NGF) (Park et al., 2000). Conversely the activation of GSK3β by Pyk2 in response to lysophosphatidic acid (LPA) contributes to neurite retraction in a neuroblastoma cell line expressing LPA1 receptor (Sayas et al., 2006). These studies in neuronal cell lines indicate that Pyk2 can participate in both neurite formation and retraction.

Studies in neurons have also outlined positive and negative effects of Pyk2 on neurites and spines. Yet it is important to mention that Pyk2 knockout mice do not display any gross defect in brain development (Giralt et al., 2017), indicating that the role of Pyk2 in this context is not major and/or that it can be replaced by FAK. Several results suggest that Pyk2 has a role downstream of integrins, especially β1-integrin, to facilitate spine formation or stability. Pyk2 together with FAK and the p130Cas/Fyn complex downstream of β1-integrin is negatively regulated by EphA4 during spine remodeling (Bourgin et al., 2007). In orbitofrontal cortex neurons, decreased β1-integrin signaling, which involves Pyk2, results in spine loss (Whyte et al., 2021). Although the role of integrins was not investigated, in Pyk2 KO mice, a decrease in spine number and PSD-95 clusters was observed in the dendrites of pyramidal CA1 hippocampal neurons *in vivo* and in culture (Giralt et al., 2017). In KO neurons in culture, PSD-95 clusters were rescued by reexpression of Pyk2, requiring its kinase activity, its autophosphorylation, and the presence of its C-terminal region (Giralt et al., 2017). In contrast rescue of spine density required the C-terminal region but neither kinase activity nor autophosphorylation. This latter observation raises the possibility that the positive role of Pyk2 on spines is independent of its kinase activity and recruitment of SFKs through autophosphorylation, but involves other properties of the molecule such as its scaffolding role.

In contrast, several reports provide evidence that Pyk2 can have a negative effect on spines. In hippocampal neurons in culture protocadherins control dendritic development by inhibiting Pyk2 inhibitory effect on small G protein Rac1 activity (Suo et al., 2012). In this study, a negative effect on spines of Pyk2 overexpression was observed. The negative regulation of Rac1 by Pyk2 downstream of protocadherins also impairs neuronal migration (Fan et al., 2018). Similarly, Pyk2 overexpression was shown to decrease spine formation through the inhibitory interaction of Pyk2 with Graf1c, a RhoA GTPase-activating protein (Lee et al., 2019). When Pyk2 is overexpressed or activated, RhoA activity is increased leading to actomyosin contraction and synapse loss (Lee et al., 2019).

Thus the studies of the role of Pyk2 in neuronal cell lines and neurons disclose opposing effects of Pyk2 on neurites and spine formation and/or maintenance. Pyk2 may participate in integrin-adhesion-triggered signaling with a positive effect on spines, whereas its overexpression or potentially its increased activity may result in a dysregulation of small GTPases Rac1 and RhoA leading to spine loss. The intriguing possibility that these two roles of Pyk2 on spines depend on different properties of the molecule will have to be further investigated. At any rate, these results indicate the requirement of tight control of Pyk2 for normal spine regulation.

### Role of Pyk2 in the Regulation of Ion Channels

**FIGURE 4:**
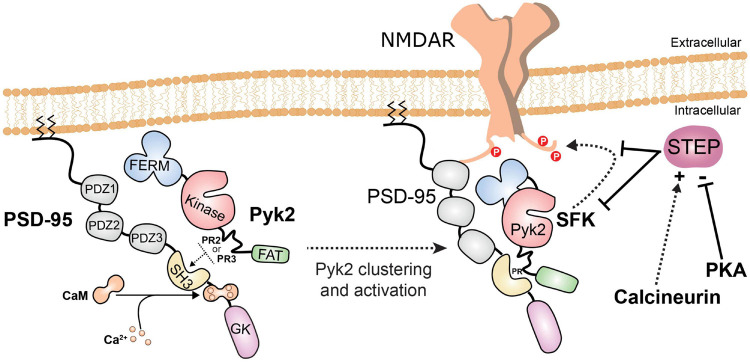
Pyk2 recruitment and activation in post-synaptic densities. When intracellular Ca^2+^ increases, Ca^2+^/calmodulin (CaM) interacts with the PSD-95 region between its SH3 and guanylate kinase-like (GK) domains (Seabold et al., 2003; Bartos et al., 2010). This makes the SH3 domain available for interaction with PR2 or PR3 motifs in Pyk2 and facilitates its enrichment in PSDs. Pyk2 can also interact with other PSD proteins (SAP102, SAPAP3) but the regulation and dynamics of these interactions have not been investigated. The binding of Ca^2+^/calmodulin to PSD-95 favors dimerization of PSD-95 and clustering and activation of Pyk2, which is in the vicinity of NMDAR. Ca^2+^/calmodulin can also bind directly to and activate Pyk2 (not shown, see Figure 2). Pyk2 and the associated SFKs, presumably mostly Fyn and Src, phosphorylate NMDAR on tyrosine. One consequence of tyrosine phosphorylation of NMDAR is to increase its surface expression. The main phosphatase which opposes these effects by dephosphorylating tyrosine residues on Pyk2, SFKs, and NMDAR is STEP. STEP is inhibited by phosphorylation by cAMP-dependent protein kinase (PKA) and indirectly activated by the Ca^2+^-activated phosphatase calcineurin.

As mentioned above (section “Pyk2 Accumulation in Post-synaptic Densities”), Pyk2 can interact with PSD proteins PSD-95, SAP102, and SAP90/PSD-95-associated protein-3, SAPAP3 (Seabold et al., 2003; Bongiorno-Borbone et al., 2005), providing a basis for its enrichment near NMDAR (Figure 4). Moreover, Pyk2 is clustered in PSDs following stimulation of NMDAR (Bartos et al., 2010). Tyrosine phosphorylation of PSD-95 itself by SFKs may play a role in the recruitment of Pyk2 (Zhao et al., 2015). PSD-95 and SAP102 are MAGUK proteins (membrane-associated guanylate kinase) so named because one of their domains is similar to guanylate kinases although it is devoid of catalytic activity. These Pyk2-binding proteins encompass several PDZ domains that can bind various post-synaptic proteins including glutamate receptors subunits, in particular those from NMDAR [see (Won et al., 2017) for a review]. Pyk2 was first identified as a component of the NMDAR complex through proteomics (Husi et al., 2000). NMDAR GluN2A and GluN2B subunits COOH-terminal region is phosphorylated at several tyrosine residues by SFKs (Tyr842, −1292, −1325, and −1387 in GluN2A and Tyr1252, −1336, and −1472 in GluN2B) [reviews in Salter and Kalia (2004); Trepanier et al. (2012); Wang et al. (2014)]. The overall effect of these phosphorylations is to increase surface expression and function of the receptor. Accordingly, several reports support the role of Pyk2 in upregulating NMDA currents. The presence of Pyk2 in the patch pipette enhances NMDA currents in hippocampal neurons, an effect that requires its kinase activity (Huang et al., 2001). The activation of Pyk2 is implicated in the upregulation of NMDAR function linked to their increased surface expression, induced by stimulation of metabotropic glutamate receptor 1 (mGluR1) (Heidinger et al., 2002) or of PACAP receptor 1 (Macdonald et al., 2005) as well as in the fast response to glucocorticoids (Yang et al., 2013). Interestingly it was recently shown that the increased number of synaptic NMDAR induced by BDNF results from the enhanced dendritic translation of Pyk2 (Afonso et al., 2019). Thus many studies indicate that Pyk2 and associated SFKs increase NMDAR function, an effect implicated in various types of neuromodulatory regulation of these receptors. In Pyk2 KO mice, phosphorylation of GluN2B Tyr1472 was decreased and the levels of NMDAR subunits and PSD-95 associated with PSDs were diminished as compared to wild-type mice (Giralt et al., 2017). It is not known whether Pyk2 phosphorylates NMDAR directly. Evidence suggests that the effects of Pyk2 are mediated by Src because the enhancement of NMDAR activity induced by Pyk2 overexpression is blocked by a Src peptide that does not act on Pyk2 while a dominant-negative mutant of Pyk2 does not affect the potentiation of NMDAR produced by Src activation (Huang et al., 2001). In neurons derived from human induced pluripotent cells, Fyn appears to be the main kinase phosphorylating GluN2B (Zhang et al., 2016). It is thus likely that Src and Fyn are downstream of Pyk2 in the regulation of NMDAR.

In contrast to the extensive study of NMDAR phosphorylation, few reports have examined the effects of tyrosine phosphorylation on AMPA receptors (AMPAR). The GluA2 subunit is phosphorylated on Tyr876 near the COOH-terminus by Src and this phosphorylation is increased in response to glutamate agonists (Hayashi and Huganir, 2004). This phosphorylation appears to facilitate activity-induced endocytosis of AMPAR. No alteration in AMPAR was observed in Pyk2 KO mice (Giralt et al., 2017). It is not known whether Pyk2 is implicated in the regulation of GluA2 Tyr876.

In addition to the regulation of glutamate receptors, Pyk2 can also contribute to the regulation of specific potassium channels. Pyk2 is involved in the downregulation of Kv1.2 channels by stimulating their endocytosis, including in response to muscarinic acetylcholine receptors (Chrm1) (Lev et al., 1995; Felsch et al., 1998; Hyun et al., 2013), leading to depolarization and opening of L-type voltage-sensitive Ca^2+^ channels in smooth muscle cells (Byron and Lucchesi, 2002). In contrast, Pyk2 has been reported to enhance the Ca^2+^-dependent activity of BK_*Ca*_ channels in non-neuronal cells (Ling et al., 2004). In neurons, these channels can be implicated in spike frequency adaptation (Ha and Cheong, 2017), but the role of Pyk2 in this context has not been investigated. Thus, the currently best-characterized effects of Pyk2 in neurons correspond to increased excitability and increased response to excitatory stimuli through the regulation of Kv1.2 channels and NMDAR.

## Pyk2 in Synaptic Plasticity

### Pyk2 in Long Term Potentiation

Long-term potentiation of synaptic transmission between Schaffer collaterals and CA1 pyramidal neurons of the hippocampus is arguably the most extensively studied form of synaptic plasticity (Nicoll, 2017). This LTP is impaired by tyrosine kinase inhibitors (O’Dell et al., 1991; Huang and Hsu, 1999) and in Fyn KO mice, but not in Src-, Yes-, or Abl-deficient mice (Grant et al., 1992). Yet, acute and specific blockade of Src prevents LTP at the same synapses (Lu et al., 1998). Because depolarization or treatment with glutamate agonists increases the phosphorylation of Pyk2 in neurons in culture and hippocampal slices (Siciliano et al., 1994; Siciliano et al., 1996), it was proposed that Pyk2 could play a role in synaptic plasticity (Girault et al., 1999a). This hypothesis has been tested in various conditions with apparently contradictory results. In CA1 pyramidal neurons of acutely prepared hippocampal slices, the presence of Pyk2 in the whole-cell recording patch pipette enhances synaptic responses through an SFK-dependent mechanism and occludes LTP induced by tetanic stimulation (two 500-ms trains of 100 Hz stimuli, separated by 10 s) (Huang et al., 2001). In this model the presence of kinase-dead Pyk2 prevents LTP induction. Disruption of the interaction between Pyk2 and PSD-95 also abolishes LTP induced by a tetanic stimulation (two 500-ms trains of 100 Hz stimuli, separated by 10 s) at synapses between Schaffer collaterals and CA1 pyramidal neurons in acute hippocampal slices (Bartos et al., 2010). In contrast, the knockdown of Pyk2 in hippocampal organotypic slices did not alter LTP induced by pairing 3 Hz stimulation (200 pulses) with postsynaptic depolarization to 0 mV (Hsin et al., 2010). In slices from Pyk2 KO mice, LTP at synapses between Schaffer collaterals and CA1 neurons induced by high-frequency stimulation (HFS, five 1-s trains of 100 Hz stimuli separated by 10 s) was abolished (Giralt et al., 2017). In a different mouse line with a similar Pyk2 KO mutation, however, the LTP induced at the same synapses by a theta-burst stimulation (TBS) protocol (10 bursts of 4 shocks at 100 Hz with an interburst interval of 200 ms) was identical to that observed in wild type mice (Salazar et al., 2019). Thus, depending on the reports the role of Pyk2 at Schaffer collaterals and CA1 pyramidal neurons appears either important or negligible. Since these various studies used different protocols to induce LTP, this question was recently re-examined by comparison of the HFS and TBS protocols in the same mouse line. In both Pyk2 constitutive KO mice and in mice in which Pyk2 deletion was induced specifically in CA1 neurons, LTP induced by HFS was impaired whereas LTP induced by TBS was unaltered, as compared to wild-type control mice (Mastrolia et al., 2021). Importantly, all the investigators who found a role of Pyk2 in LTP used an HFS induction protocol (Huang et al., 2001; Bartos et al., 2010; Giralt et al., 2017), whereas those who did not find any used either TBS (Salazar et al., 2019) or a low-frequency stimulation (Hsin et al., 2010). These combined results allow us to conclude that Pyk2 is important for LTP induced by HFS but not by TBS or low-frequency protocols.

Remarkably, the LTP that was blocked by tyrosine kinase inhibitors (O’Dell et al., 1991; Huang and Hsu, 1999), Fyn KO (Grant et al., 1992), or Src inhibition (Lu et al., 1998) was also induced by HFS. In contrast, LTP induced by 40 EPSPs evoked at 1 Hz with depolarization of the postsynaptic cell was not altered in Fyn KO as compared to wild type mice (Grant et al., 1992). This suggests that Pyk2 and tyrosine phosphorylation by SFKs are required for HFS-induced LTP and not for other types of LTP. The specific role attributed to Fyn (Grant et al., 1992) in CA1 is in agreement with the observation that Fyn is the main contributor in Pyk2 regulation in hippocampal slices (Corvol et al., 2005) although both Fyn and Src are associated with NMDAR complex (Husi et al., 2000). There is no evidence that tyrosine phosphorylation is involved in the upregulation of AMPAR (see above the discussion of AMPAR tyrosine phosphorylation in section “Role of Pyk2 in the Regulation of Ion Channels” and also below section “Pyk2 in Long Term Depression”), which are the post-synaptic substrates of LTP expression. Therefore it is likely that the contribution of Pyk2 and Fyn to LTP is linked to the phosphorylation of NMDAR and/or associated proteins under conditions of intense tetanic stimulation which triggers strictly Ca^2+^-dependent LTP (Nicoll, 2017).

The selective involvement of Pyk2 and SFKs in a specific type of LTP, observed in rather non-physiological conditions after HFS stimulation, raises the question of its biological significance. NMDAR subunit GluN2b phosphorylation at Tyr1472 is increased by HFS in conditions that induce LTP (Huang et al., 2001; Nakazawa et al., 2001), and this phosphorylation is important for LTP induced by HFS (Chen et al., 2014; Tang et al., 2015). Tyrosine phosphorylation of NMDAR increases its surface expression (Yaka et al., 2002; Prybylowski et al., 2005). In contrast, agonist stimulation, through a mechanism independent from ion fluxes but involving the glycine site, induces tyrosine dephosphorylation and endocytosis of NMDAR (Vissel et al., 2001; Nong et al., 2003). Therefore one hypothesis is that stimulation of NMDAR during HFS would induce its endocytosis and decrease the response, unless Pyk2 and associated SFKs exert the opposite effect and maintain a sufficient surface level of NMDAR for LTP induction. In support of this hypothesis, Pyk2 injection increases NMDAR currents without changing channel properties (Huang et al., 2001) and increases NMDAR surface expression (Afonso et al., 2019). Moreover, HFS-induced LTP is accompanied by an increased surface expression of NMDAR, which is blocked by an SFK inhibitor (Grosshans et al., 2002). Alternatively or in addition to this putative mechanism that would require to be experimentally tested, tyrosine phosphorylation of other Pyk2/SFK substrates, yet to be identified, might be a limiting factor in these LTP conditions. Following TBS, NMDARs are also phosphorylated on tyrosine (Pitcher et al., 2011) but additional mechanisms and signaling pathways are recruited which participate in LTP including GABA disinhibition (Larson and Munkacsy, 2015) and recruitment of the cAMP-dependent protein kinase (Abel et al., 1997; Nguyen and Kandel, 1997; Woo et al., 2003), which may exert an inhibitory role on Pyk2 activation (Alier and Morris, 2005). It is possible that in TBS-like conditions the agonist-induced NMDAR endocytosis is less pronounced than with HFS and that there is no need for a compensatory Pyk2/SFK-dependent increase in NMDAR surface expression.

### Pyk2 in Long Term Depression

In addition to its role in LTP, Pyk2 is also involved in the long-term depression (LTD) of synaptic transmission. LTD induced in hippocampal CA1 slices in organotypic culture by pairing 200 pulses at 1 Hz stimulation with depolarization of the postsynaptic cell to −40 mV was blocked by Pyk2 knockdown (Hsin et al., 2010). Similarly, LTD induced at Schaffer collaterals-CA1 synapses by 900 pulses at 1 Hz in slices from Pyk2 KO mice was abolished (Salazar et al., 2019). The molecular mechanisms of the role of Pyk2 in LTD have not been worked out in detail. GluA2 is phosphorylated on Tyr876 by SFKs in a Ca^2+^-independent manner and not directly by Pyk2 (Hayashi and Huganir, 2004). Tyr876 is located in a region that contains several tyrosine residues and is globally required for regulated AMPAR endocytosis and LTD (Ahmadian et al., 2004). Mutation of Tyr876 decreases surface expression, synaptic targeting, and agonist-induced internalization of GluA2, while phosphorylation of Tyr876 decreases its interaction with glutamate receptor-interacting protein 1/2 (GRIP1/2), indicating the existence of different modulation of basal and stimulation-induced dynamics of GluA2 (Hayashi and Huganir, 2004). Overexpression of a peptide encompassing the C-terminal sequence of GluA2 with 3 tyrosine residues prevents LTD induced *in vivo* in CA1 by paired stimuli (200 pairs at 1 Hz with an intra-pair interval of 10 ms) and LTD potentiated by stress (Fox et al., 2007). This GluA2 sequence with 3 tyrosine residues interacts with the protein BRAG2, which is a guanine-nucleotide exchange factor (GEF) for the coat-recruitment GTPase Arf6 involved in endocytosis (Scholz et al., 2010). The overall evidence suggests a role of dephosphorylation in endocytosis and LTD. mGluR-dependent LTD, induced by (*RS*)-3,5-dihydroxyphenylglycine (DHPG) is accompanied by GluA2 tyrosine dephosphorylation (Gladding et al., 2009) and is blocked by PTP inhibitors (Moult et al., 2006). Phosphorylation of Tyr876 on GluA2 prevents the activation of BRAG2 GEF activity, providing a basis for the role of dephosphorylation in endocytosis and LTD (Scholz et al., 2010). In cerebellar Purkinje cells, Tyr876 dephosphorylation by PTP-MEG recruited by GluD2-containing AMPAR is required for phosphorylation of Ser880 by PKC, leading to the replacement of anchoring proteins from GRIP to PICK1 and allowing AMPAR endocytosis during LTD (Kohda et al., 2013). Thus all these results support a role of GluA2 dephosphorylation, and not phosphorylation, in LTD and cannot account for the requirement of Pyk2. Interestingly, Pyk2 is known to be activated downstream of transient receptor potential melastatin 2 (TRPM2) in non-neuronal cells (Belrose and Jackson, 2018), and Trpm2 is a channel important for NMDAR-dependent LTD at CA3-CA1 synapses (Xie et al., 2011), but the role of Pyk2 in connection with TRPM2 has not been investigated in the hippocampus. Besides a partial contribution of its kinase activity, it has also been proposed that the scaffolding properties of Pyk2 are important for its role in LTD (Hsin et al., 2010), but the involved partners have not been identified. Thus, although it is clear that LTD is altered when Pyk2 is absent (Hsin et al., 2010; Salazar et al., 2019), the precise underlying molecular mechanisms accounting for this role are not yet known.

### Pyk2 in Intrinsic Excitability Plasticity

The regulation of Kv1.2 by SFKs and Pyk2 can play a role in the regulation of intrinsic excitability plasticity. In CA3 pyramidal neurons, a conditioning train of 20 action potentials (APs) at 10 Hz causes a persistent reduction in the input conductance and an acceleration of the AP onset time, corresponding to LTP of intrinsic excitability (LTP-IE) (Hyun et al., 2013). It depends on back-propagating APs to distal apical dendrites and the presence of Kv1.2 channels. LTP-IE is prevented by the inhibition of Kv1.2 endocytosis or of protein tyrosine kinase activity (Hyun et al., 2013). Although Kv1.2 endocytosis requires its phosphorylation, which is controlled by Pyk2 (Lev et al., 1995; Felsch et al., 1998), the role of Pyk2 in LTP-IE could not be directly confirmed due to interfering effects of Pyk2 knockdown in that preparation (Hyun et al., 2013). In conclusion, Pyk2 is involved in LTP induced by HFS, in LTD, and probably in LTP-IE, but the details of its mechanism of action in these various conditions are not fully elucidated.

## Pyk2 in Learning and Memory

The role of Pyk2 in behavior has been investigated using Pyk2-deficient mice. Pyk2 KO mice display a deficit in the spontaneous alternation and the novel object location (NOL) tests (Giralt et al., 2017). These tests explore short-term spatial memory after a single brief training session. Spontaneous alternation evaluates the ability of the animal to remember the previously explored arm of a Y maze 2 h after training. NOL measures the preference for exploring the object displaced among two identical ones, 24 h after a first exposure. In both tests visual cues are provided by geometrical patterns around the apparatus. A similar deficit in NOL was observed in mice with an AAV-induced deletion of Pyk2 in the dorsal hippocampus CA1 region (Giralt et al., 2017). The deficit in NOL was reproduced in other batches of mice from the same KO line and was rescued by targeted bilateral re-expression of Pyk2 in neurons of dorsal hippocampus CA1 (Mastrolia et al., 2021). NOL is a sensitive test that requires the integrity of the dorsal hippocampus [see (Aggleton and Nelson, 2020) for a recent review]. In contrast, the novel object recognition test (NOR) is a single-session training test similar to NOL except that instead of displacement of an object it is replaced by a different one without external visual cues. This test that does not explore spatial memory and involves several brain regions and neurotransmitter systems (Dere et al., 2007), was not altered in Pyk2 KO mice (Giralt et al., 2018; Salazar et al., 2019). Moreover, the spatial memory evaluated in the Morris water maze, a paradigm dependent on the dorsal hippocampus but based on repeated trial sessions and possibly modulated by a stressful component (Cunningham et al., 2009) was also unaltered in Pyk2-deficient mice (Salazar et al., 2019). Thus, it appears that Pyk2 deficit alters only relatively weak forms of spatial memory induced by a single brief exposure and involving dorsal hippocampus, whereas this impairment is overcome by recruitment of other brain regions and/or by repeated training. This contribution in memory contrasts with the role of Pyk2 in synaptic potentiation which is unveiled only in HFS-induced LTP, in response to a non-physiological type of intense stimulation, but not in a more physiological protocol of induction (see above). This contrast illustrates the absence of simple one-to-one correspondence between specific forms of LTP in slices and memory alterations, in spite of the widely acknowledged role of synaptic plasticity in learning and memory (Gallistel and Balsam, 2014). It is also apparent that other changes such as intrinsic plasticity are likely to contribute to memory processes (Titley et al., 2017), in which the role of Pyk2 remains to be fully explored both at the level of ion channel modulation and through its possible contribution to transcriptional regulations.

## Pyk2 in Brain Diseases

### Epilepsy

Given the activation of Pyk2 following stimulation of glutamate receptors, neuronal depolarization, and increased intracellular free Ca^2+^, it is not surprising that its phosphorylation on Tyr402 and other residues is increased following electroconvulsive shock (Jeon et al., 2001), more strongly than that of FAK (Kang et al., 2004). Tyrosine phosphorylation of NMDAR and PSD-associated Pyk2 increases in the hour following pilocarpine-induced status epilepticus and decreases subsequently (Niimura et al., 2005). Accordingly, neuronal pPyk2-immunofluorescence was decreased as compared to control mice 6 h after kainic acid-induced seizures, but, interestingly, appeared in activated microglia at 72 h (Tian et al., 2000). These results indicate the existence of early and transient activation of Pyk2 in neurons following seizures and a delayed activation in microglia. Microglial cells are the brain-resident form of macrophages, a cell type in which Pyk2 is known to play an important role (Baruzzi et al., 2008; Lee et al., 2021). The importance of inflammation and microglia is increasingly acknowledged in epilepsy, in which it has been proposed that epileptic seizures and inflammatory mediators form a vicious positive feedback loop (Sanz and Garcia-Gimeno, 2020). Thus it will be interesting to explore whether activation of Pyk2 not only in neurons but also in microglia, is involved in the pathogenic consequences of seizures and whether its inhibition might be beneficial.

### Ischemia

Another condition in which strong stimulation of glutamate receptors occurs is brain ischemia. Pyk2 is rapidly enriched in PSDs following global transient brain ischemia in the rat (Cheung et al., 2000), reminiscent of the clustering of Pyk2 at PSDs induced *in vitro* by glutamate or NMDA (Bartos et al., 2010). Following focal cerebral ischemia pPyk2 immunoreactivity increases in neurons within 1 h and 24–72 h later in microglia around the necrotic infarcted area (Tian et al., 2000). Ischemia/reperfusion increases tyrosine phosphorylation of NMDAR subunits GluN2a and GluN2b (Cheung et al., 2000). The increase in tyrosine phosphorylation of GluN2a, Pyk2, and Src is reduced by pretreatment with inhibitors of the L-type voltage-gated calcium channel (Liu et al., 2003). Tyrosine phosphorylation of Src and Pyk2, but not their translocation to PSDs, is decreased by a PKC inhibitor (Cheung et al., 2003). Moreover, chronic lithium is neuroprotective during ischemia and inhibits the phosphorylation of Pyk2 at Tyr402 and Src at Tyr416 (Ma et al., 2004). Knockdown of Pyk2 decreases GluN2a tyrosine phosphorylation (Liu et al., 2005), and knockdown of PSD-95 decreases Pyk2 phosphorylation and neuronal death (Hou et al., 2005). Interestingly, the role of Pyk2 in ischemia or hypoxia is not restricted to the brain and has also been investigated in several non-neuronal cell types including in cardiomyocytes (Dougherty et al., 2004; Hart et al., 2008; Yan et al., 2015; Bibli et al., 2017a,b; Zhang et al., 2018; Miller et al., 2019). Overall the evidence indicates that Pyk2 is activated in ischemia/reperfusion models in the brain and in other organs, due to Ca^2+^ influx and possibly to redox imbalance. In neurons, the triggering mechanisms include the activation of glutamate receptors and depolarization-induced Ca^2+^ influx, clustering of Pyk2 to PSDs, activation of Pyk2 and SFKs, and NMDAR phosphorylation, through a mechanism that is at least in part dependent on PKC. This in turn may contribute to excitotoxicity by enhancing NMDAR function and possibly contributing to other toxic effects as in non-neuronal cells. Thus, Pyk2 inhibition would appear as a good candidate for therapies aiming to protect the brain after ischemia.

### Huntington’s Disease

Huntington’s disease (HD) is characterized by motor and behavioral symptoms as well as cognitive decline leading to death. HD is an autosomal dominant alteration caused by the expansion of a CAG repeat in the human huntingtin gene (*HTT*) resulting in an extended poly glutamine sequence in the N-terminal part of the huntingtin protein which alters its stability and function [review in Saudou and Humbert (2016)]. Wild-type huntingtin and Pyk2 interact with the same SH3 domain of PSD-95 (Sun et al., 2001; Seabold et al., 2003). Poly glutamine extension alters Htt binding to PSD-95 and increases tyrosine phosphorylation of GluN2B by SFKs possibly facilitating neurotoxicity (Sun et al., 2001; Song et al., 2003). Pyk2 levels are decreased in the hippocampus of patients with HD and in R6/1 mice (Giralt et al., 2017), which overexpress an N-terminal fragment of human Htt with expanded glutamine (Mangiarini et al., 1996). The decrease in Pyk2 expression is likely to result, at least in part, from transcriptional alterations since its mRNA is also diminished [(Runne et al., 2008) and al Massadi et al. in preparation]. R6/1 mice display synaptic alterations in the hippocampus reminiscent of those in Pyk2 KO mice (Giralt et al., 2017). Pyk2 overexpression in the hippocampus of these mice improved their memory deficits, spine pathology, and PSD-95 mislocalization, but not LTP alterations. It is thus likely that the Pyk2 deficit in the hippocampus contributes to some aspects of cognitive impairments in HD, which can potentially be improved by Pyk2 increased expression. Similar decreases in Pyk2 protein and mRNA levels are found in the putamen of HD patients and the striatum of R6/1 and R6/2 mouse models (al Massadi et al. in preparation). However, it is not known whether this deficit contributes to the phenotype. Interestingly, the levels of STEP, the phosphatase that inactivates Pyk2, are also decreased in patients and mouse models of the disease, and its phosphorylation on serine is increased, indicating inhibition of STEP activity (Saavedra et al., 2011). This was interpreted as a possible compensatory mechanism that could increase neuronal resistance to excitotoxicity. In support of this hypothesis, genetic deletion of STEP delayed the onset of motor dysfunction and prevented the appearance of cognitive deficits in R6/1 mice, and acute pharmacological inhibition of STEP with TC-2153 improved cognitive function in these mice (Garcia-Forn et al., 2018). Although STEP has other targets that can mediate these effects, including ERK, it is tempting to propose that decreasing the negative control exerted by STEP on Pyk2 and the associated SFKs contributes to the beneficial effects of STEP inhibitors and reinforces their therapeutic interest.

### Alzheimer’s Disease

Several lines of evidence implicate Pyk2 at multiple levels in Alzheimer’s disease (AD) and AD mouse models. The first line of evidence comes from human genetic studies. A large genome-wide association (GWAS) meta-analysis identified the *PTK2B* gene as a locus associated with late-onset AD (Lambert et al., 2013). This finding was replicated in several other studies (Beecham et al., 2014; Jiao et al., 2015; Wang et al., 2015; Nettiksimmons et al., 2016; Lawingco et al., 2021; Schwartzentruber et al., 2021). Moreover, in AD patients, *PTK2B* appears to be associated with hippocampal sclerosis (Beecham et al., 2014), disease progression (Wang et al., 2015), and cognitive decline (Nettiksimmons et al., 2016). Until now, no alteration in the coding sequence of PTK2B has been reported and the variants are more likely to be linked to modifications of expression. One single nucleotide polymorphism (SNP), rs28834970C, associated with an increased risk of AD (Lambert et al., 2013) was found to be associated with increased *PTK2B* mRNA expression in monocytes of healthy subjects (Chan et al., 2015). Another SNP associated with increased risk of AD, rs2279590, is located in an enhancer near the *PTK2B* gene and the risk allele increases expression of several genes including *PTK2B* (Padhy et al., 2017). Moreover, a recent transcriptome study found an association between AD and increased *PTK2B* expression in monocytes (Harwood et al., 2021). Together these observations suggest that late onset AD risk is associated with increased expression of Pyk2 protein in monocytes, and probably in tissue-resident macrophages such as microglia, and possibly in other cell types. Interestingly, genes coding for proteins of the p130Cas/BCAR1 family that interact with Pyk2 and/or FAK (Deneka et al., 2015), including *CASS4* (Lambert et al., 2013) and *NEDD9* (Li et al., 2008) are also associated with an increased risk of late-onset AD, but it is not known whether this corresponds to their participation in the same pathophysiological mechanisms in the disease.

**FIGURE 5:**
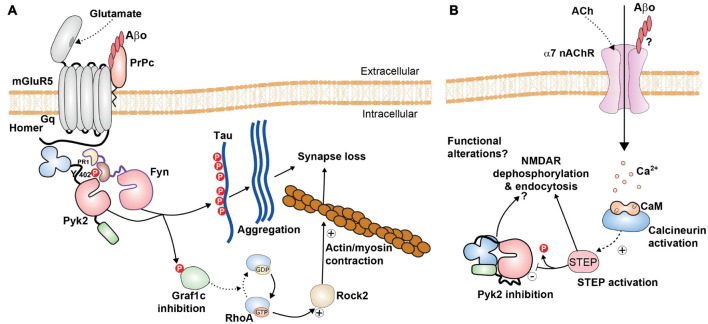
Possible roles of Pyk2 in Alzheimer’s disease. **(A)** Pyk2 is likely to be a component of the amyloid cascade mechanisms. Pyk2 can be associated with the complex between mGluR5 and Prp_*c*_, associated with homer. The binding of Aβ oligomers (Aβo) to Prp_*c*_, a glycosylphosphatidylinositol-anchored extracellular protein, impairs normal signaling of mGluR5 but triggers the activation of Fyn and Pyk2. These kinases phosphorylate Tau and may thus facilitate its aggregation and the formation of neurofibrillary tangles. Pyk2 also phosphorylates the RhoA GTPase Graf1c, inhibiting its activity, increasing active RhoA/GTP, resulting in activation of Rock2 and actin/myosin contraction that can contribute to synaptic alterations [see references in the text and (Brody and Strittmatter, 2018)]. **(B)** These pathways can potentially play a different role, possibly in different neurons or at different localizations or different times in the same neurons, with activation of STEP, for example following Ca^2+^ entry through α7 nicotinic acetylcholine receptor (α7nAChR) and activation of calcineurin. This could result in tyrosine dephosphorylation of Pyk2 and NMDAR, decreased NMDAR surface expression, and functional synaptic alterations. The balance between the mechanisms depicted in **(A,B)** is not known and may depend on experimental models and conditions. Additional roles of Pyk2 are possible but not explored, including in microglial cells (not shown).

Alzheimer’s disease hallmarks include amyloid plaques, neurofibrillary tangles, and glial responses as well as “negative” lesions including neuronal and synaptic loss (Serrano-Pozo et al., 2011). Extracellular amyloid plaques are comprised of β-amyloid peptide Aβ_1–42_, the amyloidogenic and toxic fragment of the amyloid precursor protein APP, while neurofibrillary tangles are aggregates of hyperphosphorylated microtubule-binding protein Tau. Data from several laboratories indicate multiple levels at which Pyk2 could be involved in the course of the disease. The group of Strittmatter provided strong evidence that Pyk2 participates in a cascade triggered by oligomeric Aβ (Aβo), the major toxic form of Aβ [review in Brody and Strittmatter (2018); Figure 5A]. The cellular prion protein (PrPc), which is a receptor of Aβo, associates with mGluR5 and by its intermediate with Homer1b/c, Pyk2, and Ca^2+^/calmodulin-dependent protein kinase II (Haas et al., 2016, 2017; Haas and Strittmatter, 2016). Aβo slightly increases Pyk2 phosphorylation while AZD0530, an inhibitor of Fyn, decreases Pyk2 and GluN2b tyrosine phosphorylation (Kaufman et al., 2015) as expected from the decreased Pyk2 phosphorylation observed in Fyn KO mice (Corvol et al., 2005). This inhibitor improves several histological and behavioral parameters in APPSwe/PSEN1ΔE9 mice [bearing 2 human mutations responsible for familial AD, APP KM670/671NL (Swedish) and presenilin-1 (PSEN1) ΔE9 (Jankowsky et al., 2004)]; Kaufman et al. (2015). Aβo disrupts glutamate’s ability to regulate Pyk2 and Ca^2+^/calmodulin-dependent protein kinase II signaling through mGluR5, whereas Pyk2 is activated by Fyn (Haas and Strittmatter, 2016). In APPSwe/PSEN1ΔE9 mice lacking Pyk2, the deleterious effects of Aβo on synaptic plasticity (TBS-induced LTP) and behavioral alterations are prevented (Salazar et al., 2019). Pyk2 KO also improves dendritic spine loss induced by Aβo and this effect may result from the alleviation of inhibition by Pyk2 of Graf1c, a RhoA GTPase-activating protein (Lee et al., 2019). This counterintuitive positive effect of deletion of Pyk2 on spine density reflects the ambivalent function of Pyk2 in spine formation and maintenance discussed above (section “Pyk2 in Neurite and Spine Formation and Maintenance”). It is likely that in APPSwe/PSEN1ΔE9 mice, the increased stability gained by deleting Pyk2 overcomes the possible negative effects linked to the absence of Pyk2. Overall, these results argue in favor of a deleterious role of Pyk2 following its release from mGluR5 complex and its phosphorylation by Fyn, due to Aβo interaction with PrPc (Brody and Strittmatter, 2018).

Pyk2 is also linked with tauopathy since Tau is a substrate of Fyn (Lee et al., 2004; Bhaskar et al., 2005, 2010) and Pyk2 (Li and Gotz, 2018). Pyk2 co-localizes with hyperphosphorylated Tau in the brain of AD patients and in the Tau transgenic pR5 mouse model (Kohler et al., 2013; Dourlen et al., 2017). Phosphorylation by Fyn, which can be activated by Pyk2 (Li and Gotz, 2018), promotes the somatodendritic accumulation of Tau, whereas this protein is normally located in axons (Li and Gotz, 2017). Pyk2 can also activate GSK3, which phosphorylates Tau [review in Hooper et al. (2008)]. Moreover, drosophila FAK, the single ortholog of FAK and Pyk2 in this species, is a modulator of Tau toxicity in a fly eye model of neurodegeneration (Dourlen et al., 2017). Taken together, these results suggest that Pyk2 could contribute to tauopathy by directly and indirectly modifying Tau phosphorylation at several sites, although it does not seem to be necessary for the prion-like spreading of pathological human Tau microinjected in mouse brain (Nies et al., 2021). Thus, Pyk2 is a good candidate for linking Aβ and tau pathology in the framework of the amyloid cascade hypothesis (Figure 5A).

Other evidence sheds a different light on the role of Pyk2 and suggests it may be more complex than anticipated based on its contribution to the pathogenic cascades (Figure 5B). It was first proposed that the scaffolding protein Ran-binding protein 9 (RanBP9), which is markedly increased in AD brains, promotes Aβ_1–42_ generation by scaffolding APP/BACE1/LRP complexes together and accelerating APP endocytosis through a mechanism that involves disruption of Pyk2/paxillin signaling (Woo et al., 2012). When 5XFAD mice, which express transgenic human APP and PSEN1 with several mutations [APPSwe, I716V (Florida), V717I (London) and PSEN1 M146L, L286V (Oakley et al., 2006)] were crossed with Pyk2 KO mice, their behavioral and histological phenotypes were not improved, except for a small decrease in plaque number (Giralt et al., 2018). In 5XFAD mice, Pyk2 Tyr402 phosphorylation levels in the hippocampus were reduced and overexpression of Pyk2 in this brain region rescued autophosphorylated Pyk2 levels and improved synaptic markers and performance in several behavioral tasks, despite a slight increase in plaque number (Giralt et al., 2018). The presence of a potentially toxic proteolytic fragment of Src was observed in 5XFAD and Pyk2 KO mice, which was corrected by Pyk2 viral expression (Giralt et al., 2018). In further support of the positive role of Pyk2, its overexpression in hippocampal neurons in a microfluidic culture system also protected these neurons from Aβ_1–42_ toxicity (Kilinc et al., 2020). These results suggest that in some conditions a functional deficit of Pyk2 could play a role in AD model. The possible positive effects of increasing Pyk2 expression/phosphorylation are in agreement with the effects of inhibition of STEP, its main phosphatase. Aβ enhances STEP activity, through a mechanism that involves α7 nicotinic acetylcholine receptor and calcineurin (PP2B) activation leading to dephosphorylation and decreased surface expression of NMDAR (Snyder et al., 2005). Moreover, STEP levels are increased in an AD mouse model and in human cortical samples (Chin et al., 2005; Kurup et al., 2010). The phenotype of 3xTg AD mouse model carrying mutated forms of human APPSwe, Tau P301L, and PSEN1 M146V (Oddo et al., 2003) is improved when they are crossed with STEP KO mice (Zhang et al., 2010). Behavioral improvement is also observed following treatment with a pharmacological inhibitor of STEP and is accompanied by increased phosphorylation of Pyk2 (Xu et al., 2014). Genetic reduction or pharmacological inhibition of STEP also increases synaptic connectivity in cell cultures and the triple transgenic AD mouse models (Chatterjee et al., 2021). These various lines of evidence suggest that increased STEP activity and diminished tyrosine phosphorylation pathways, including those mediated by Pyk2, could have a negative effect in the context of AD.

An additional level of complexity comes from the role of Pyk2 in microglial cells that are an important player in AD (Saez-Atienzar and Masliah, 2020). In a culture system, Pyk2 is involved, downstream of PKC, in the secretion of neurotoxic factors by microglia stimulated by Aβ (Combs et al., 1999). TRPM2 is a ROS-sensitive calcium channel expressed in neurons, astrocytes, and microglia, which has been implicated in many neurological diseases (Belrose and Jackson, 2018). In monocytes (the circulating form of macrophages), TRPM2 activates a Pyk2/ERK pathway leading to the generation of chemokines and exacerbating inflammation (Yamamoto et al., 2008). This signaling pathway is involved in microglial activation by Aβ_1–42_ and the subsequent generation of cytotoxic tumor necrosis factor-α (TNFα), with Pyk2 playing a positive feedback role on the TRPM2 channel (Alawieyah Syed Mortadza et al., 2018). As described above data in human subjects suggest that PTK2B variants associated with increased risk of AD lead to a higher expression of Pyk2 in monocytes and possibly in tissue-resident macrophages (Chan et al., 2015; Harwood et al., 2021). Although the existence of an increased Pyk2 expression in microglia has not been tested, it is tempting to speculate that it could contribute to the microglial/neuroinflammation arm of AD pathology. Under this hypothesis Pyk2 in microglia could be an interesting therapeutic target.

In conclusion, Pyk2 is in a position to play multiple roles in AD mouse models and possibly in late-onset AD in human patients. In neurons, Pyk2 in interaction with Fyn is a mediator of Aβ toxicity and can link Aβ effects with Tau aberrant phosphorylation, making it a potential key player in the amyloid cascade. On the other hand, a functional deficit in Pyk2, possibly related to an enhanced STEP activity, may contribute to some aspects of the functional deficits in mouse models of AD. It remains to be established which of these two opposing mechanisms are predominant depending on the experimental model and conditions and whether they play a role in human patients. Finally, the possible contribution of Pyk2 in microglia to the pathogenic mechanism of AD needs to be further investigated. It is tempting to suggest that depending on the genetic variants of *PTK2B*, slight changes in its expression regulation may favor one arm or the other of these multiple aspects of Pyk2 role. These questions have practical importance for future therapeutic developments since selective inhibitors of Pyk2 are being developed and could constitute useful therapeutic tools, as well as, but in the opposite direction, inhibitors of STEP, which indirectly increase Pyk2 function.

### Parkinson’s Disease

The protein which is the main component of Lewy bodies and plays a key role in the neurodegeneration in Parkinson’s disease, α-synuclein, is phosphorylated by SFKs, including Fyn, on several residues, mostly Tyr125 (Ellis et al., 2001; Nakamura T. et al., 2001). This phosphorylation is increased in response to hyperosmotic shock through activation of Pyk2 (Nakamura et al., 2002) and inhibited by a Pyk2/RAFTK-associated protein (PRAP) that binds to Pyk2 PR3 motif through its SH3 domain (Takahashi et al., 2003). Phosphorylation of α-synuclein at Tyr125 is a priming event for phosphorylation of Ser129 by protein kinase CK1, a site predominantly phosphorylated in Lewy bodies (Kosten et al., 2014). However, Tyr125 phosphorylation has been proposed to have a neuroprotective role in a drosophila model of synucleinopathy (Chen et al., 2009b). This protective effect may result from the prevention by Tyr125 phosphorylation of the sequestration in Lewy bodies by α-synuclein of phosphoinositide-3 kinase enhancer L (PIKE-L), a GTPase abundantly found in nerve termini, which has neuroprotective effects (Kang et al., 2017). No alteration of Pyk2 has been reported in Parkinson’s disease but STEP levels were shown to be elevated in brains from patients with Parkinson’s disease and STEP61, the membrane-associated isoform of STEP, is upregulated in Parkinson’s disease (Kurup et al., 2015). These results could suggest impaired tyrosine phosphorylation in Parkinson’s disease due to increased STEP. However, the role of Fyn in Parkinson’s disease is generally thought to be negative, and potential therapeutic interest of its inhibitors has been proposed [see (Angelopoulou et al., 2020) for a recent review].

### Psychiatric Disorders and Drug Addiction

A few studies associated Pyk2 with chronic stress damage and depressive disorders. In the lateral septum, Pyk2 phosphorylation decreases following stress exposure, and enhancing Pyk2 expression prevents the active avoidance deficit caused by exposure to inescapable shock, suggesting an antidepressant role (Sheehan et al., 2003). In the rat prefrontal cortex, imipramine, an antidepressant drug that blocks the reuptake of monoamine neurotransmitters, activates a pathway involving Pyk2 activation and ERK phosphorylation (Zalewska et al., 2016). In major depression in humans and in a mouse chronic stress model, a decrease in the nuclear pore protein NUP62 transcript was reported (Kinoshita et al., 2014). NUP62 is phosphorylated by Pyk2, facilitating its dissociation from the nuclear pore, and pTyr402-Pyk2 accumulates in the perinuclear space of CA3 hippocampal neurons following chronic stress in the rat (Kinoshita et al., 2014). Therefore the authors proposed that Pyk2 activation and the subsequently decreased function of NUP62 participate in the phenotype possibly by altering the dendritic arbor complexity. Pyk2 has also been implicated in a mouse model of depressive state induced by chronic unpredictable mild stress (CUMS) (Montalban et al., 2019). In Pyk2 KO mice, anxiety and anhedonia induced by CUMS were less pronounced than in wild-type littermates whereas another phenotype (struggling in the forced swimming test) was not altered. This improvement of behavioral consequences of CUMS observed in constitutive KO mice was mimicked by a bilateral targeted deletion of Pyk2 in the amygdala (Montalban et al., 2019). A possible explanation of the beneficial effect of Pyk2 deletion in this model is that it specifically prevents the stress-induced synaptic plasticity in the amygdala, which leads to the depressive-like phenotype.

Another common brain disorder extensively studied in animals is addiction. Chronic exposure to cocaine was shown to increase Pyk2 levels in primate nucleus accumbens (Freeman et al., 2001) and rat frontal cortex (Freeman et al., 2002) leading to the idea of a possible implication of Pyk2 in addiction. A neuroprotective role of Pyk2 has been described in rats receiving moderate ethanol preconditioning (Mitchell et al., 2016). This preconditioning induces neuroprotection against oxidative stress possibly involving a Pyk2/NMDAR pathway leading to the elevation of an antioxidant protein, peroxiredoxin 2 (Prx2). The acute locomotor effects of cocaine injection are decreased in Pyk2 KO mice as compared to wild-type (de Pins et al., 2020). Targeted deletions suggest that this effect results from the absence of Pyk2 in the dopamine D1 receptors-expressing neurons of the nucleus accumbens. In contrast, behavioral alterations that result from cocaine effects on neuronal plasticity, locomotor sensitization, and conditioned place preference were normal in Pyk2 mutant mice (de Pins et al., 2020). A recent study in mice showed that cocaine self-administration triggers a loss of dendritic spines on orbitofrontal cortex excitatory neurons (Whyte et al., 2021). The authors provide evidence that β1-integrin plays a key role in stabilizing spines and that the behavioral effects of cocaine are counterbalanced by Pyk2 overexpression. These results suggest that Pyk2 participates in spines stabilization downstream of β1-integrin regulating drug self-administration and possibly other effects. Thus the few studies on the role of Pyk2 in depression and addiction models suggest that it may play a role by supporting plasticity in specific brain regions, with either negative behavioral consequences when it involves amygdala in stress-induced depression, or positive ones when it takes place in orbitofrontal cortex in addiction.

### Pyk2 in Astrocytes and Glioma

Astrocytes have critical importance in the nervous tissue and are directly involved in synaptic function. Pyk2 is expressed in astrocytes where it can be activated by endothelin-1 (Cazaubon et al., 1997), stromal cell-derived factor-1, also known as SDF-1 (Bajetto et al., 2001), thrombin (Wang and Reiser, 2003), and angiotensin II (Clark and Gonzalez, 2007), and is upstream of the ERK pathway. In many cell types, FAK and Pyk2 play a role in cell migration, by facilitating cytoskeleton remodeling and cell adhesion dynamics [see (Schaller, 2010) for a review]. In particular, Pyk2 regulates actin dynamics in non-neuronal cells such as macrophages, fibroblasts, and osteoclasts (Okigaki et al., 2003; Lim et al., 2008b; Li et al., 2017). Pyk2 involvement in astrocytes migration is supported by the slower migration of Pyk2 KO astrocytes as compared to wild-type astrocytes after physical lesion and their impaired actin re-polymerization after treatment with the actin-depolymerizing drug latrunculin B (Giralt et al., 2016). This effect possibly involves Pyk2 interaction in astrocytes lamellipodia with gelsolin, an actin-binding, severing, and capping protein (Giralt et al., 2016) as shown in osteoclasts (Wang et al., 2003). Pyk2 may also play a role in anti-oxidant responses of astrocytes as indicated by its involvement in the arachidonic acid-induced increase in heme oxygenase-1 (HO-1) (Lin et al., 2018).

Beyond these roles in normal astrocytes, Pyk2 implication in astroglial tumors has attracted a lot of attention and the detailed description of the results is beyond the scope of this review. Pyk2 has been implicated in various cancer types in which it constitutes an interesting therapeutic target [review in Zhu et al. (2018)]. Protein levels of Pyk2 are increased in invasive glioblastoma cells (Hoelzinger et al., 2005) and expression levels are associated with the migration capacity of human glioblastoma cells whereas FAK activity is linked to proliferation (Lipinski et al., 2003). siRNA knockdown of Pyk2 further confirmed the central role of Pyk2 in the migration of glioblastoma cells (Lipinski et al., 2005; Rolon-Reyes et al., 2015). The interaction between Pyk2 and MAP4K4 (mitogen-activated protein kinase-kinase-kinase-kinase 4) was shown to be decisive in this function (Loftus et al., 2013). Interestingly, melatonin could be a potential therapeutic molecule able to reduce Pyk2 activation and, thus, inhibit tumor cell migration/invasion (Xu et al., 2015). In neuroblastoma, Pyk2 may also play an important role as it was shown that TRPM2 expression increases the viability of neuroblastoma through a pathway involving Pyk2 activation (Hirschler-Laszkiewicz et al., 2018). Taken together, these results indicate that Pyk2 could be a potential target for glioma and neuroblastoma therapies.

## Pyk2 Inhibitors

Pyk2 like FAK has been implicated in various cancers in which it contributes to increase cell viability or migration by activating various oncogenic pathways and the expression level of these two proteins is often associated with poor prognosis [reviews in Sulzmaier et al. (2014); Zhu et al. (2018)]. Therefore much effort has been devoted to the identification of small molecules capable to inhibit these kinases and several compounds are undergoing clinical trials in cancer. The inhibitors usually target the ATP binding site and because of the high degree of structural conservation between FAK and Pyk2, most bind to and inhibit the two kinases. However, several compounds have been reported to display a certain degree of selectivity for Pyk2 including PF-4618433 (Han et al., 2009) and PF-719 (Tse et al., 2012). However, given the diversity and importance of FAK functions, this low selectivity is likely to hamper the clinical exploration of such inhibitors in conditions other than cancers with poor prognosis. The design of compounds targeting other regions of Pyk2 or FAK could lead to more specific inhibitors or molecules interfering only with specific properties of these multifunctional proteins with multiple partners (Walkiewicz et al., 2015). This is an open area for research, which could also lead to compounds enhancing some functions of Pyk2. In this respect, the only current way to pharmacologically enhance Pyk2 function, albeit non-specifically, is to inhibit the phosphatases responsible for its dephosphorylation, especially STEP in brain neurons (Xu et al., 2014; Chatterjee et al., 2021). When specific compounds are identified and the role of Pyk2 in the various neurological or psychiatric diseases discussed above is better understood, the exploration of their therapeutic applications will be of great interest.

## Conclusion

Pyk2 is highly expressed and has important cellular and physiological functions in the central nervous system. Its presence is not necessary for the basic functioning of the brain that is grossly normal in Pyk2 KO mice, which grow and breed normally in animal facility conditions. In addition and in contrast to FAK, Pyk2 levels in the rodent brain increase dramatically in the post-natal period pointing to a more specific role in the late development or in adult brain than during early development. Convergent evidence shows that Pyk2 is involved in specific aspects of synaptic functions and plasticity. However Pyk2 signaling intervenes in multiple pathways, and has therefore multiple functions with in some cases opposing effects. Thus, depending on models and experimental conditions it can facilitate or inhibit neurite and dendritic spine formation. Pyk2 is also a component of the complex signaling machinery that regulates NMDAR in the post-synaptic region. As such it appears necessary for LTD and specific types of LTP, as well as for single-trial spatial memory. Multiple lines of evidence also point to the role of Pyk2 in pathological conditions. This role appears to be multifaceted with sometimes apparently opposite contributions to the pathology in mouse models. Pyk2 activation may contribute to the neurotoxic consequences of epilepsy and ischemia. Pyk2 levels or activity may be deficient in some neurodegenerative conditions such as in HD or AD, where this deficit can contribute to alterations in synaptic functions and behavior that are improved by expression of Pyk2 or inhibition of the major Pyk2 phosphatase STEP. In contrast, Pyk2 appears to be a key component in the cascade of events triggered by Aβ in models of Alzheimer’s disease where it may provide a link between amyloid toxicity and tauopathy. In addition, Pyk2’s enrichment in cells of the macrophage lineage including in activated microglia raises the possibility of its contribution to the neuroinflammatory component of various conditions including epilepsy, ischemia, and neurodegenerative diseases. In cancer in general and in glioblastoma in particular Pyk2 contributes to tumor cells’ malignancy, particularly by facilitating their mobility, indicating the potential therapeutic benefit of Pyk2 inhibitors in combination with other drugs. Since in neurological and psychiatric conditions the role of Pyk2 may include positive as well as deleterious aspects, it is important to precisely evaluate its contribution, including ultimately in humans, to balance the potential benefit of its pharmacological inhibition or its possible enhancement, including through the manipulation of tyrosine phosphatases such as STEP.

## Author Contributions

All authors listed have made a substantial, direct and intellectual contribution to the work, and approved it for publication.

## Conflict of Interest

The authors declare that the research was conducted in the absence of any commercial or financial relationships that could be construed as a potential conflict of interest.

## Publisher’s Note

All claims expressed in this article are solely those of the authors and do not necessarily represent those of their affiliated organizations, or those of the publisher, the editors and the reviewers. Any product that may be evaluated in this article, or claim that may be made by its manufacturer, is not guaranteed or endorsed by the publisher.
